# Osteonecrosis of the jaw in an AIDS patient: a case report

**DOI:** 10.1186/s12981-015-0049-8

**Published:** 2015-04-30

**Authors:** Yusuke Zushi, Kazuma Noguchi, Kuniyasu Moridera, Kazuki Takaoka, Hiromitsu Kishimoto

**Affiliations:** Department of Oral and Maxillofacial Surgery, Hyogo College of Medicine, 1-1, Mukogawa-cho, Nishinomiya, Hyogo 663-8501 Japan

**Keywords:** Osteonecrosis of the jaw, AIDS, HAART

## Abstract

We report a rare case of osteonecrosis of the jaw following necrotizing gingivitis in a Japanese AIDS patient. Intraoral examination showed exposed necrotic bone in the left mandible and spontaneous loss of teeth. This patient was successfully treated with highly active anti-retroviral therapy combined with minimally invasive surgical procedures to remove the osteonecrosis of the jaw.

## Background

Oral complications of human immunodeficiency virus (HIV) infection, such as oral candidiasis, hairy leukoplakia, Kaposi’s sarcoma, and herpes simplex labialis, are sometimes the first sign of the infection and often indicate progression to AIDS [1,2]. Cases with definitive diagnosis of AIDS with osteonecrosis of the jaw are extremely rare in Japan and other developed countries. We present herein a rare case of osteonecrosis of the jaw in a 37-year-old Japanese AIDS patient treated with minimally invasive surgery.

## Case report

A 37-year-old Japanese man complaining of discomfort in the left mandibular molar area was referred to our department. Six months earlier, he had noticed pain on swallowing and loosening of the teeth in the left mandibular molar area. Medical and family histories were non-contributory. The patient had a history of smoking and alcohol consumption, but no drug allergies. General examination showed the patient was underweight (body mass index (BMI), 14.2 kg/m^2^), and he complained of a 5-kg reduction in weight over the preceding 2 months.

**Figure 1:**
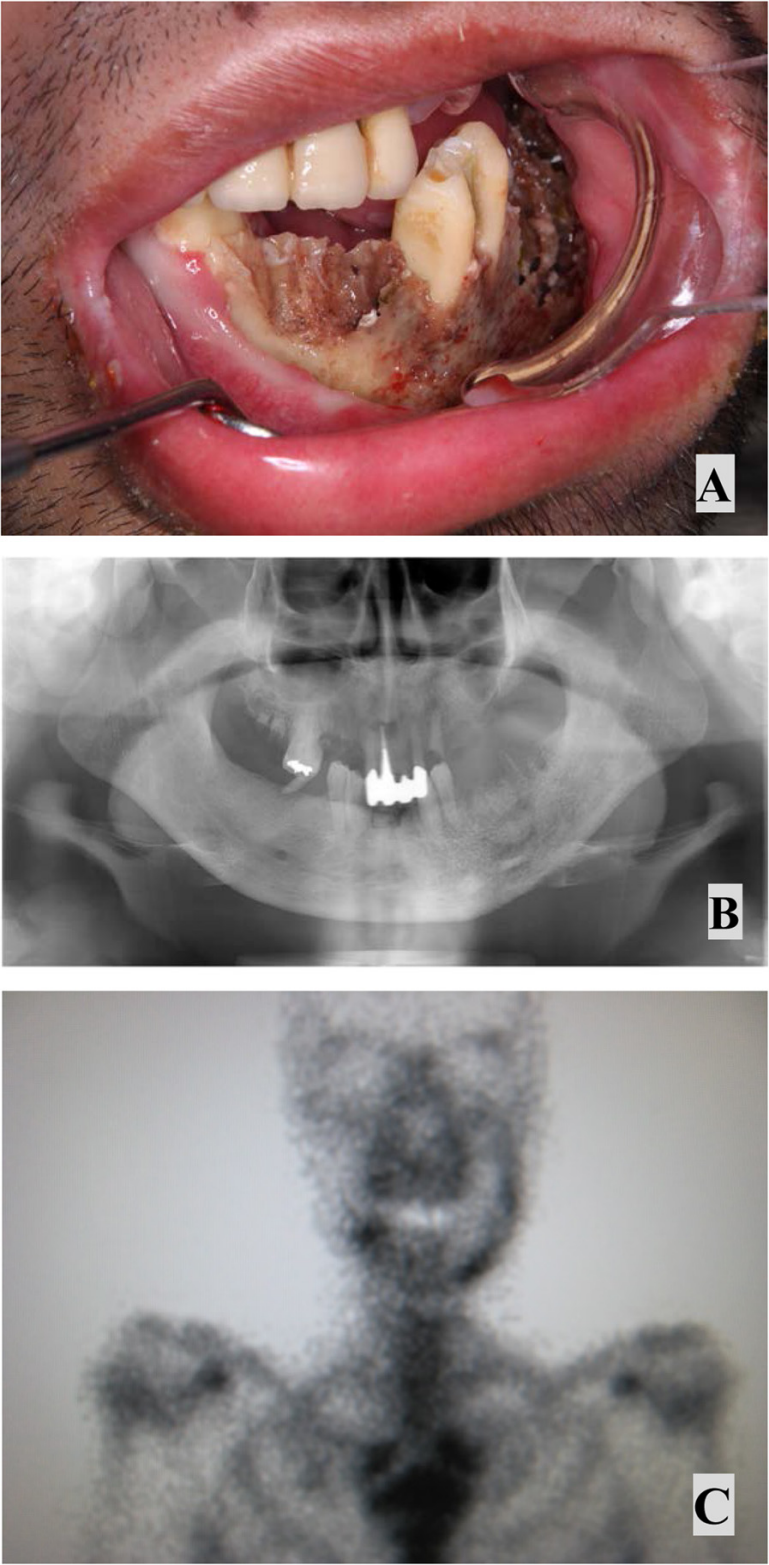
**Findings on admission. A)** Left mandible with denudation of bone, spontaneous loss of the teeth, necrotizing gingivitis and stomatitis. **B)** Orthopantomography show ill-defined, irregular bony destruction in the left mandible. **C)** Gallium-67 citrate scintigraphy shows abnormal uptake in the left mandible.

**Figure 2:**
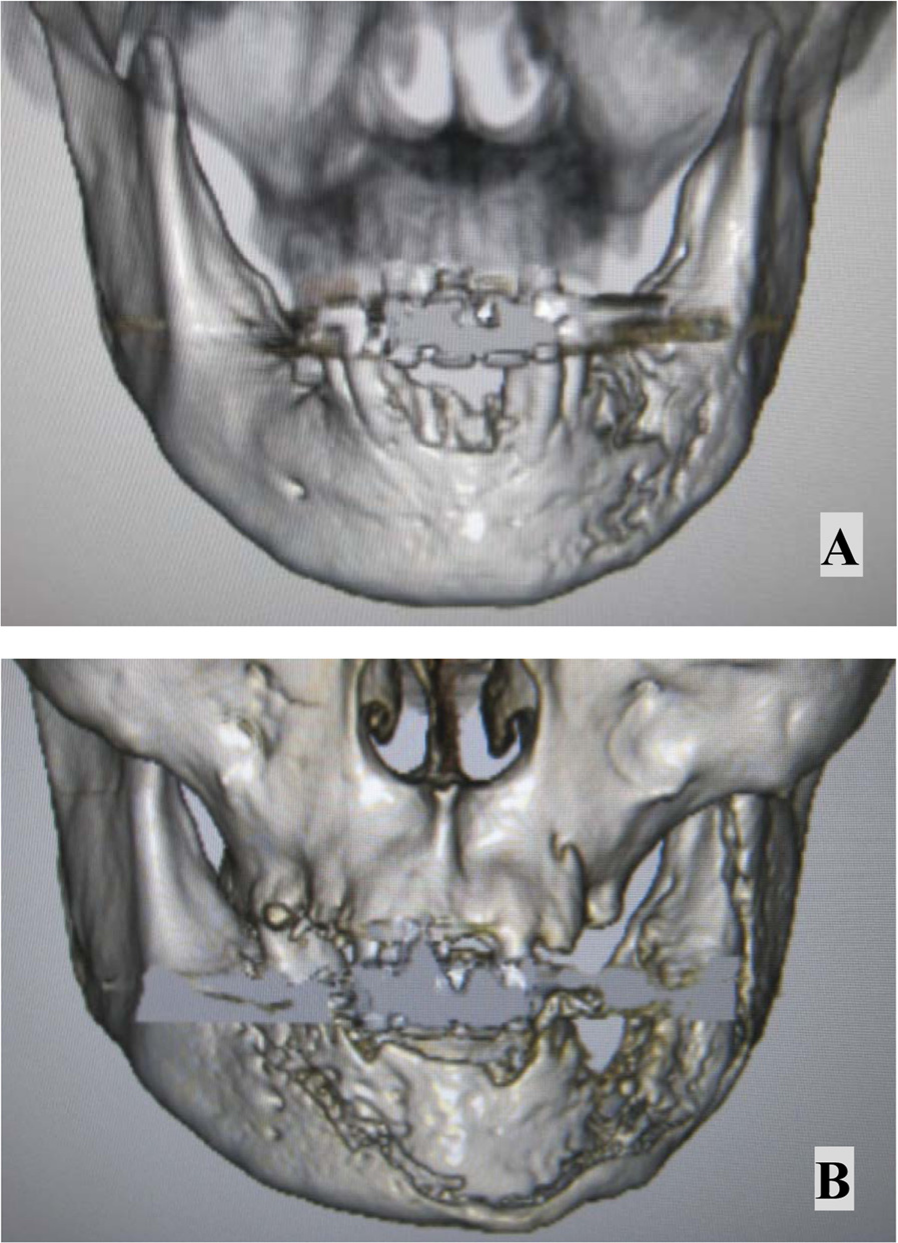
**Changes in appearance on 3D-CT from on admission. (A)** to the 2-month follow-up examination **(B)**. **A)** 3D-CT showed ill-defined, irregular destruction of cortical bone in the left mandible on admission. **B)** Sequestra of the mandible were seen as radiopaque areas resembling “islands of bone” at the 2-month follow-up.

Extraoral examination showed facial asymmetry caused by swelling of the left cheek. No paresthesia was noted over the lower lip. Lymph node swelling was palpable bilaterally in the submandibular regions, but lymph nodes felt normal to the touch. Intraoral examination showed necrosis of the left mandible with exposed bone, a missing right mandibular canine tooth, necrotizing gingivitis and stomatitis that had destroyed the buccal mucosa and floor of the mouth (Figure 1A). Orthopantomography showed ill-defined, irregular bony destruction on the lower alveolar ridge extending from the left canine tooth to the left second molar, with floating teeth at the right canine and first premolar in the right mandible (Figure 1B). Gallium-67 citrate scintigraphy showed abnormal uptake in the left mandible (Figure 1C). Computed tomography (CT) showed a bone-destroying lesion occupying the left alveolar ridge, extending from the mucosal tissue of the left cheek to the floor of the mouth. Multiple enlarged lymph nodes were observed in the cervical area. Three-dimensional (3D)-CT showed destruction of cortical bone in the left mandible (Figure 2A). Results of chest radiography and electrocardiography were within normal limits.

On first impression, malignant tumor such as lymphoma or multiple myeloma was strongly suspected from the clinical findings and imaging results. The patient was hospitalized immediately, and the gingival lesion was biopsied. Histological examination showed no evidence of malignancy, but did reveal severe inflammatory granulation with lymphoid aggregates. We considered the possibility of osteomyelitis of the mandible and administered clyndamycin at 2400 mg/day as antimicrobial therapy. The patient was malnourished on admission, and nutritional status improved with the administration of electrolytes and fluids. Daily irrigation of the mandibular lesion with saline was started to reduce contamination, and sloughed tissue was removed.

Hematological examination showed reductions in the white blood cell count (3,950/μL; normal, 4,000-9,000/μL), red blood cell count (3.07 × 10^6^/μL; normal, 3.80-5.30 × 10^6^/μL), hemoglobin (8.9 g/dL; normal, 12.0-16.5 g/dL), and hematocrit (28.1%; normal, 35.0-45.0%). Serology yielded negative results for HBV and HCV, and results of liver and renal function tests were unremarkable. Rouleaux formation of red blood cells was observed, but blood levels of M protein were within normal limits. Venereal examination showed positive results, and HIV testing was performed.

HIV enzyme-linked immunosorbent assay testing showed positive results. The patient was found to be anti-HIV-antibody positive. The patient then reported he was homosexual. His CD4 count was 78/μL, and the plasma HIV-RNA load was 2.5 × 10^5^ copies/mL.

HAART was started with emtricitabine at 200 mg/day, tenofovir disoproxil fumarate/abacavir at 300 mg/day, and raltegravir potassium at 1600 mg/day to treat AIDS, followed by ethambutol at 1000 mg/day, rifampicin at 300 mg/day, clarythromycin at 800 mg/day, and ciprofloxacin at 1200 mg/day for the non-tuberculous mycobacterial infection.

One month after initiating HAART, the HIV-RNA load markedly decreased (from 2.5 × 10^5^ copies/mL to less than 44 copies/mL), but the CD4 count was 78/μL. For 3 months after initiation of HAART, CD4 counts showed no dramatic changes.

At the 2-month follow-up, necrotic bone of the mandible was seen as radiopaque areas resembling “islands of bone” on both orthopantomography and 3D-CT (Figure 2B). The sequestrum of the mandible subsequently separated from living bone, with a layer of new bone (termed involucrum) forming around the necrotic bone.

**Figure 3:**
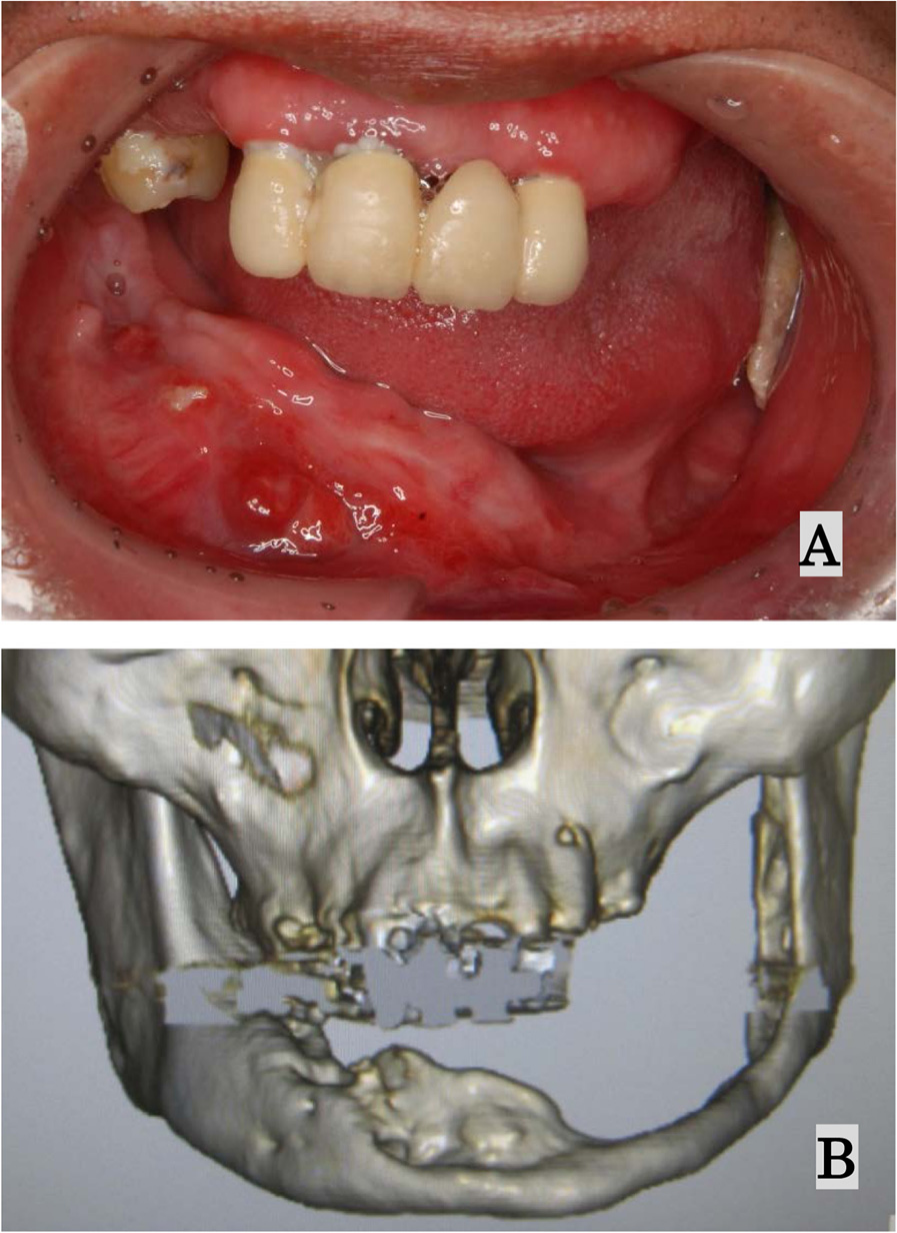
**Appearance at the 6-month follow-up examination.** In the absence of radical surgery, spontaneous exfoliation of sequestrum is observed, showing spontaneous expulsion through the mucosa. **A)** Extensive scarring is seen around the mandibular defect. **B)** On 3D-CT, the living bone is seen to be very thin in the left mandible.

**Figure 4:**
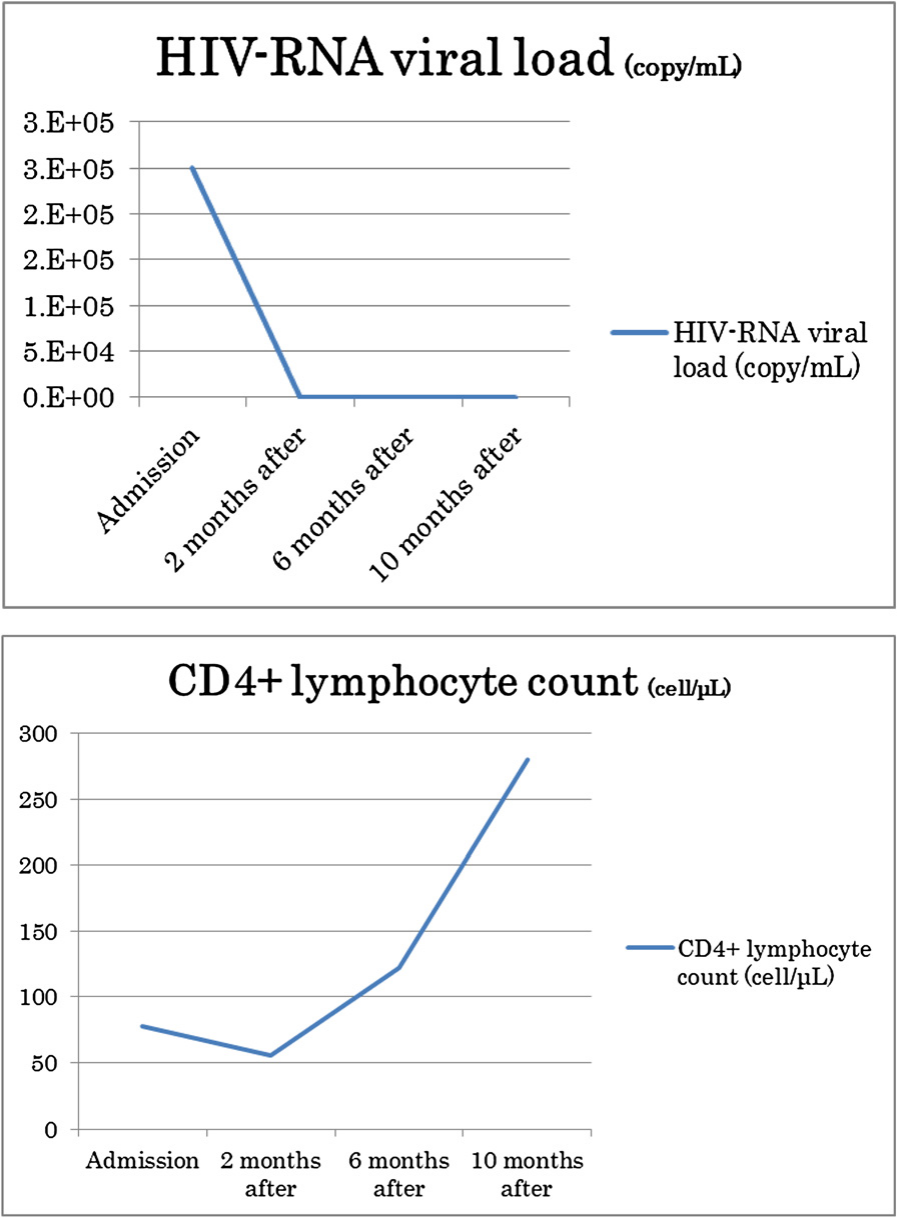
**Treatments and changes in HIV-RNA viral load and CD4 count.** Viral load decreased from 2.5 × 10^5^ copies/mL to <44 × 10^5^ copies/mL with treatment. During the first 10 months after treatment, CD4+ lymphocyte count increased from 78 cells/μL to 280 cells/μL.

At the 6-month follow-up examination, without performance of any radical surgery, spontaneous exfoliation of the necrotic bone was observed, partially resorbed by granulation tissue or spontaneously expelled through the mucosa; in the latter instances, necrotic bone was removable through less-invasive surgical procedures. Extensive scarring was seen around the mandibular defect (Figure 3A). CT showed that living bone was very thin in the left mandible, with a risk of pathological fracture due to a significant volume of bone loss (Figure 3B). Ten months after starting HAART, the CD4+ lymphocyte count had increased from 78 cells/μL to 280 cells/μL (Figure 4). The patient regained more than 10 kg of body weight compared to status on admission and his general condition was markedly better (BMI increased from 14.2 kg/m^2^ to 19.1 kg/m^2^). With continued antiretroviral therapy, the patient has shown good immunological recovery, with no signs of relapse as of one and a half years after starting HAART.

## Discussion

Oral disorders occur in approximately 60% of HIV/AIDS cases and may present as a wide range of lesions, notably fungal, viral, and bacterial infections and malignant neoplasms such as Kaposi’s sarcoma [2]. Cases involving definitive diagnosis of AIDS from a finding of osteonecrosis of the jaw are very rare in Japan. We initially suspected malignant tumor or osteomyelitis of the mandible, and AIDS was not considered at all. Due to the strong suspicion of osteomyelitis of the mandible, antimicrobial therapy was initiated at the same time.

HAART was started after obtaining results from HIV testing, and the HIV-RNA load subsequently decreased markedly from 2.5 × 10^5^ copies/mL to less than 44 copies/mL. CD4 counts, however, showed little change within 3 months. HIV-positive patients with CD4+ lymphocyte counts below 200 cells/ml are well known to be severely immunocompromised and HIV-positive patients with a viral load greater than 10,000 copies/ml show active viremia [2]. It is currently impossible to determine the relative contributions of local treatment, improved nutritional status and improved immunological status following HAART to the arrest of the subacute phase of osteonecrosis of the mandible seen within 2–6 months of follow-up. If not adequately treated, the condition of this patient might well have developed to noma (cancrum oris). Noma represents a sequence of odontogenic infections characterized by an irregular pattern of rapidly spreading necrosis of the gingiva, mucosa, fascia, muscles, and skin. In developed countries, noma is extremely rare and affects only patients with severe immunosuppression or blood dyscrasia [3,4]. By contrast, noma persists in the poorest developing countries, mostly typically in Africa. If not adequately treated, patients with noma will die, usually from secondary infection, septicemia, severe dehydration and malnutrition, and rarely hemorrhage [5-8]. Noma is suggested to frequently start as necrotizing gingivitis [3], and the treatment regimen for this precursor disease has to include antibiotic treatment appropriate to anaerobic infections, frequent mouth-washing and gentle tooth cleaning to remove and aerate the necrotic anaerobic pabulum. However, depending on the extent of intra-oral damage, debridement of necrotic bone, extraction of mobile teeth and dental rehabilitation may be required.

In the present case, whether the living bone in the left mandible needed reinforcement with a reconstruction plate to minimize the risk of fracture is highly controversial. If pathological fracture occurred due to reduced bone strength in the mandible, consideration would need to be given to subsequent reconstruction, such as a reconstruction plate, or autogenous bone grafting. We think that avoiding reconstruction may be the most practical option from the perspective of quality of life (QOL) at present. However, the use of HAART is associated with loss of bone mineral density independent of HIV infection [9,10]. This decrease in bone density is suggested to be a direct effect of protease inhibitors on bony remodeling or an indirect effect on vitamin D metabolism [11]. Furthermore, initiation of HAART can trigger “immune reconstitution inflammatory syndrome” (IRIS), an inflammatory cascade that can decrease BMD and place patients at even greater risk of osteoporotic fracture [12]. We assume that HAART use may have had some bearing on adverse events and fracture risk in this patient.

The patient currently has a good appetite, and is able to chew quite well despite extensive scarring around the defect. His QOL remains moderately good.

## Consent

Written informed consent was obtained from the patient for publication of this case report and the accompanying images.

## Abbreviations

AIDS: Acquired immunodeficiency syndrome
HAART: Highly active antiretroviral treatment
HIV: Human immunodeficiency virus
HBV: Hepatitis B virus
HCV: Hepatitis C virus
CT: Computed tomography
3D-CT: 3-dimensional computed tomography
BMI: Body mass index
QOL: Quality of life
IRIS: Immune reconstitution inflammatory syndrome
BMD: Bone mineral density
